# Influence of Nickel Content and Heat Treatment Parameters on Kinetics of Crystallisation, Magnetic Properties and Brittleness of Nanocrystalline Fe-Ni-B Alloys Obtained by Ultra-Rapid Annealing with Joule Heating

**DOI:** 10.3390/ma19102157

**Published:** 2026-05-21

**Authors:** Jarosław Ferenc, Zofia Czyżewska, Maciej Kowalczyk, Krzysztof Sielicki, Dariusz Oleszak

**Affiliations:** Faculty of Materials Science and Engineering, Warsaw University of Technology, ul. Wołoska 141, 02-507 Warsaw, Poland; zofia.czyzewska.stud@pw.edu.pl (Z.C.); maciej.kowalczyk@pw.edu.pl (M.K.); krzysztof.sielicki2@pw.edu.pl (K.S.)

**Keywords:** nanocrystalline alloys, metallic glass, crystallization, ultra rapid annealing, Joule heating, soft magnetic materials, glass transition temperature

## Abstract

Metallic glasses can be transformed into nanocrystalline–amorphous alloys via controlled crystallisation with fast nucleation and slow grain growth. This can be achieved either through appropriate chemical composition of amorphous precursors or by applying ultra-rapid annealing (URA). Typically, heating between preheated copper blocks is used to ensure the URA conditions. In this work, ribbons were heated by an electric current flowing along their length, and the temperature was monitored using pyrometers. The investigated alloys were Fe_86-x_Ni_x_B_14_ (at. %), where *x* = 4, 6 or 10. Properly adjusted isothermal annealing at 380–410 °C for 1–20 s induced crystallisation, with the nanocrystalline bcc-Fe(Ni) phase occupying 0–55% of the volume. With increasing annealing time, the coercive field increased from 9 A/m in the amorphous state to 25 A/m and 17 A/m for *x* = 4 and *x* = 10, respectively. Transmission electron microscopy confirmed that samples annealed at higher temperatures for shorter times exhibited smaller grain sizes compared to those annealed at lower temperatures for longer times, which resulted in improved magnetic softness. An increase in nickel content reduced coercivity, improved ductility, and offered a wider window for the choice of annealing temperature.

## 1. Introduction

Magnetically soft alloys with amorphous–nanocrystalline structures have been intensively studied since 1988, when Yoshizawa et al. developed the Fe–Cu–Nb–Si–B alloy (Finemet) [1]. Initially, their variants were obtained through compositional modifications and conventional heat treatment by isothermal annealing in a furnace for tens of minutes. Nanocrystals formed from the amorphous matrix due to the presence of non-magnetic elements that enhanced nucleation and suppressed grain growth. In 2011, Herzer et al. introduced rapid annealing by sliding amorphous ribbons along preheated steel blocks to increase the heating rate [2]. A similar approach was used by Pradeep et al. [3], where stationary pieces of ribbon were pressed between preheated copper blocks for a short time. In both cases, the crystallised alloys contained the grain-refining elements. These studies demonstrated that nucleation is highly sensitive to heating rate, leading to increased nuclei density. The concept of rapid heating was further extended by Suzuki et al. in 2017 to Fe-Ni-B alloys without grain-refining elements, thereby increasing the fraction of ferromagnetic atoms. This approach enhanced saturation induction while maintaining small grain size and low coercivity [4]. The addition of nickel reduces magnetocrystalline anisotropy, *K*_1_, in iron alloys, giving rise to a reduction in coercivity [5]. Small grain size further improves the magnetic softness by lowering the random-averaged magnetocrystalline anisotropy, <*K*_1_>, in nanocrystalline alloys [6]. Therefore, reduction in grain size is very important in improving soft magnetic properties by manipulating the microstructure of nanocrystalline alloys, while the addition of nickel affects the intrinsic magnetic properties. Li et al., by application of a copper block technique and modification of nickel content, obtained nanocrystalline alloys with bcc-Fe(Ni) and fcc-Fe(Ni), with both of these phases as the nanocrystalline component, which led to them obtaining an average grain size of 4.2 nm and a coercivity as low as 2.6 A/m [7]. Ultra-rapid annealing has been extensively investigated to improve soft magnetic properties and to better understand crystallisation under extreme heating rates and short annealing times. Various rapid heating techniques have been explored, including laser pulses [8,9], microwaves [10] and Joule heating (pulses or continuous) [11,12]. In the present work, Joule heating is employed, enabling temperature monitoring and control of the predetermined thermal cycle, as well as monitoring of ribbon extension upon tension to determine glass transition temperature. Amorphous ribbons of Fe_86-x_Ni_x_B_14_ alloys were partially crystallised to create nanocrystalline alloys and investigate the crystallisation kinetics of the process and the effect of nickel content on microstructure, magnetic properties, and brittleness.

## 2. Materials and Methods

Fe_86-x_Ni_x_B_14_ alloys, where *x* = 4, 6 or 10 (at. %), further referred to as Ni4, Ni6 and Ni10, respectively, were made by arc melting of electrolytic iron with 99.9% purity, nickel with 99.99% purity and an iron–boron master alloy of “nuclear-grade” purity. The choice of chemical composition was based on our earlier research, which proved that the addition of nickel improves the magnetic properties of nanocrystalline alloys and widens the window of useful annealing temperature where the crystallisation process is easy to control, and the alloys with nickel were the most promising for combining good magnetic softness and saturation magnetization [13]. Amorphous ribbons were produced in the Institute of Non Ferrous Metals, Łukasiewicz Research Network, Gliwice, Poland, by melt-spinning of liquid alloys (1340 °C) onto a copper drum rotating at 800 rev./min speed. The density of amorphous ribbons was measured using the Micromeritic Accupyc II 1340 helium pycnometer. The cross-section of each ribbon was calculated from the mass, length and density data, and was used to calculate resistivity and tensile stress. The cross-section of the ribbons was equal to 0.1512, 0.1420 and 0.1205 mm^2^ for Ni4, Ni6 and Ni10, respectively. The width of the ribbons was about 8 mm. The ribbons were cut into 153 mm long sections and mounted in a custom-built ultra-rapid annealing system. The external parts of the sections were clamped in holders with electric contacts, leaving 100 mm of net length of the heat-treated sample. High temperature was achieved by Joule heating from a current flowing along the ribbon, while temperature was measured using a pyrometer and controlled via a proportional–integral–derivative (PID) microcontroller, where the feedback signal was obtained from pyrometers. In addition to the controlling function, the microcontroller collected data on electric current, voltage drop and length of the sample. The main current is adjusted by a MOSFET transistor, whose gate is opened with the signal from the microcontroller [14]. Thermal cycles consisted of continuous heating, isothermal soaking, and controlled or free cooling. During processing, electrical resistance (four-probe method) and sample length were monitored continuously. In the first part of this study, continuous heating experiments (30–500 °C/s) were used to determine glass transition and crystallisation temperatures. Isothermal annealing was then performed at 100 °C/s with holding times of 0–20 s (0 s corresponding to immediate cooling).

Structural analysis was performed by X-ray diffraction (Rigaku Miniflex II). Crystalline volume fraction and grain size were determined by peak deconvolution and the Scherrer formula, using the Jade software for XRD pattern processing. Hysteresis loops were recorded in the home-built hysteresis loop tracer based on the concept described in [15]. The accuracy of coercive field determination is 0.7 A/m. To measure brittleness, samples after heat treatment were bent to 180° and slowly squeezed by parallel surfaces, and the distance between the plates at the moment of fracture (*S*_f_) was recorded. From these measurements, the coefficient of ductility, expressed as

*λ* = *g*/(*S*_f_-*g*), (1)

was calculated, as proposed in [16]. The microstructure of the nanocrystalline alloys was observed with the JEOL JEM 1200EX transmission electron microscope (TEM), with the accelerating voltage of 120 kV. Thin foils for TEM observations were obtained by ion polishing in a GATAN PIPS system, model 691, with an accelerating voltage of 5.1 kV and grazing angle from 8 to 4 degrees.

## 3. Results and Discussion

### 3.1. Heat Treatment

#### 3.1.1. Continuous Heating

During continuous heating, two parameters were monitored: electrical resistance and sample length. Resistance reflects crystallisation progress, as the crystalline phase has lower resistivity than the amorphous phase. Thus, the onset of resistance decrease indicates the onset of crystallisation. Sample length, measured under slight tension, reflects both thermal expansion and viscous flow when the amorphous ribbon transitions into a supercooled liquid.

**Figure 1:**
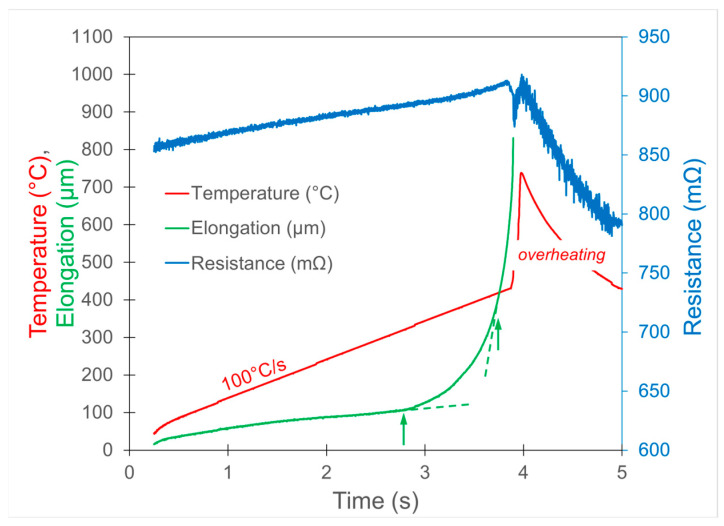
Time dependence of temperature, resistance and elongation of the as-quenched Fe_80_Ni_6_B_14_ alloy subjected to continuous heating at the rate of 100 °C/s.

For all compositions and heating rates, the qualitative behaviour of temperature, resistance, and elongation was similar; see Figure 1 as an example. Resistance increased linearly with temperature until crystallisation onset, followed by a decrease due to the formation of bcc-Fe(Ni). Elongation under tension of 50 MPa initially followed linear thermal expansion and then deviated due to viscous flow. This effect is attributed to the transition from the solid state of rigid metallic glass to the supercooled liquid state, which deforms easily upon tension. In this experiment, it is difficult to indicate a certain temperature at which a solid alloy turns into a liquid form, as the glass transition occurs over a temperature range; the temperature of glass transition was defined using a range from the onset to accelerated elongation (in Figure 1, these are indicated by green arrows).

After crystallisation onset, temperature rapidly increases because the heat of crystallisation is released very quickly and this leads to immediate self-overheating. During the annealing to form the nanocrystalline–amorphous structure, this has to be avoided, because magnetically hard iron borides are formed in the second stage of crystallisation, and soft magnetism is lost.

**Figure 2:**
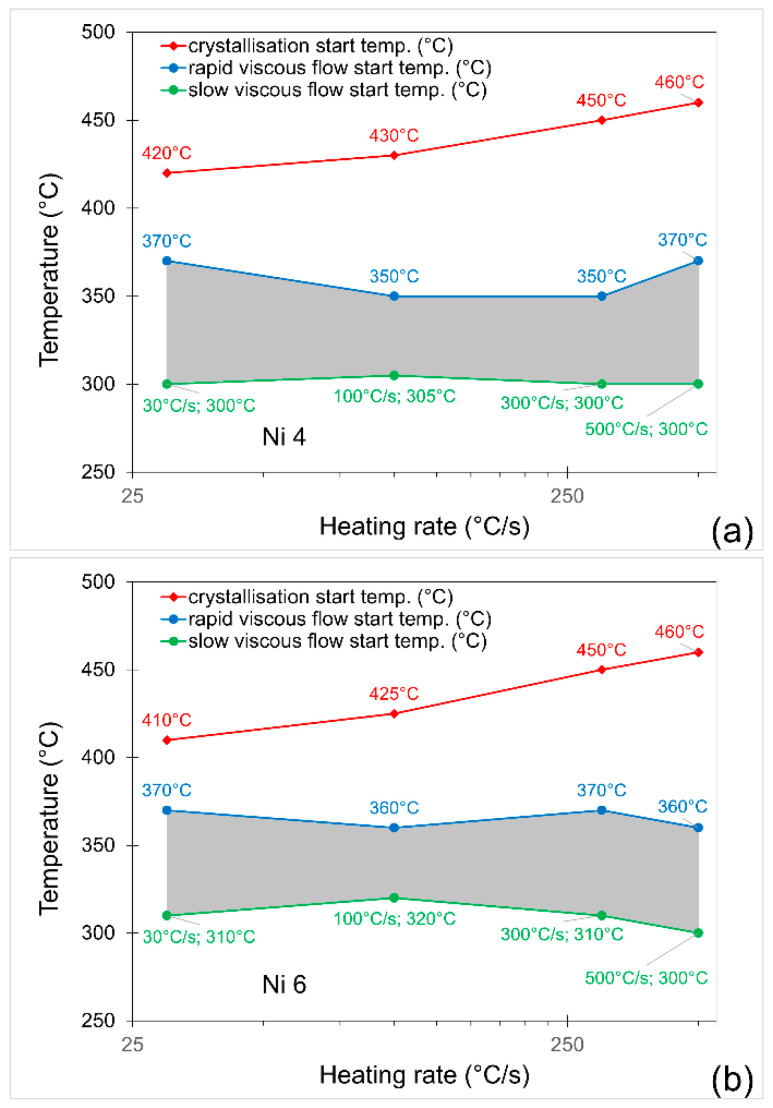

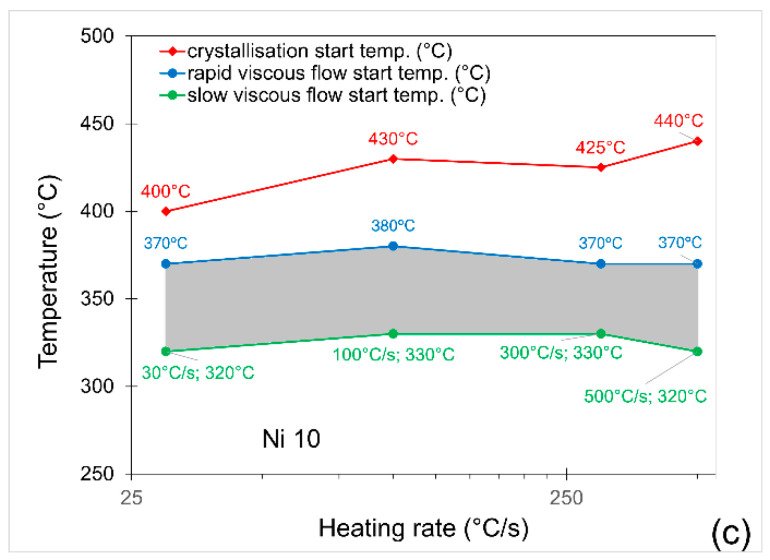
Dependence of crystallisation onset temperature and glass transition temperature band on the heating rate for (**a**) Fe_82_Ni_4_B_14_, (**b**) Fe_80_Ni_6_B_14_ and (**c**) Fe_76_Ni_10_B_14_ alloys.

Figure 2 shows the dependence of onset crystallisation temperature, *T*_x_ (upper line), and the range of glass transition temperature, *T*_g_ (grey band), on the heating rate. For all the investigated alloys, the onset crystallisation temperature increases with the increase in heating rate, as expected. However, for the glass transition temperature band, there is no such clear dependence—the *T*_g_ band remains stable. The most important conclusion from Figure 2 is that the first stage of crystallisation starts above the glass transition temperature, i.e., in the supercooled liquid region. This favours high nucleation rates and increases the probability of the formation of a nanocrystalline structure [17].

#### 3.1.2. Isothermal Annealing

**Figure 3:**
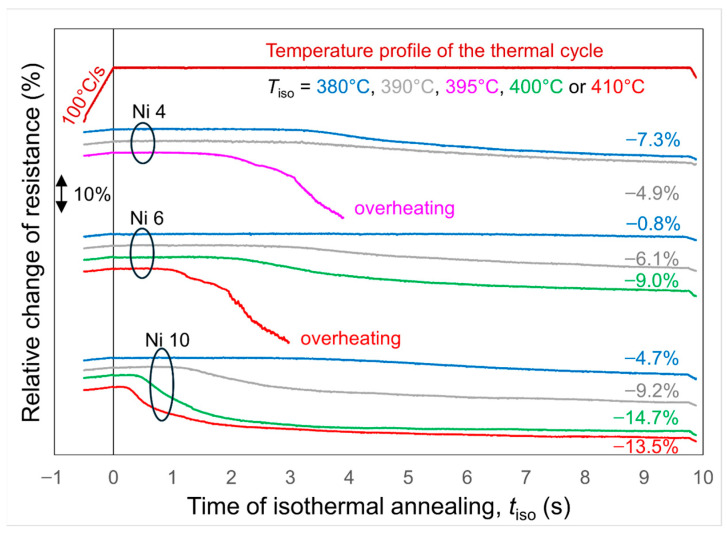
Time dependence of resistance of the as-quenched Fe_86-x_Ni_x_B_14_ alloys subjected to isothermal annealing for 10 s.

Isothermal annealing was carried out with an invariable heating and cooling rate of 100 °C/s. For each of the alloys, annealing at 370 °C for 10 s did not provoke crystallisation, so it was not taken into further consideration. The crystalline phase appeared after isothermal annealing at temperatures of 380 °C and higher. The dependence of resistance on the time of the isothermal stage is presented in Figure 3. At the beginning of the isothermal stage, the incubation period takes place when no crystallisation occurs, so the resistance of the samples is constant. Its length (*τ*_0_) depends on nickel content and on the temperature of annealing. Table 1 indicates the estimated duration of incubation time. At higher annealing temperatures the values of *τ*_0_ are shorter. A higher content of nickel also reduces the incubation time, although this dependence is not monotonous—the incubation times for Ni6 are longer than for other alloys. The shorter incubation time observed for higher Ni content suggests that there are more nuclei ready to grow, and crystallisation is quantitatively detected. If the addition of Ni facilitates the formation of supercritical nuclei, it may be expected that the final microstructure will be finer, and the nanocrystalline–amorphous alloys will be magnetically softer.

Once crystallisation starts, the relative reduction in resistance is subtle at lower annealing temperatures, and becomes more pronounced at higher annealing temperatures. This drop in resistance is directly related to the increase in the crystalline phase volume fraction.

### 3.2. Structure Investigations

#### 3.2.1. X-Ray Diffraction

**Figure 4:**
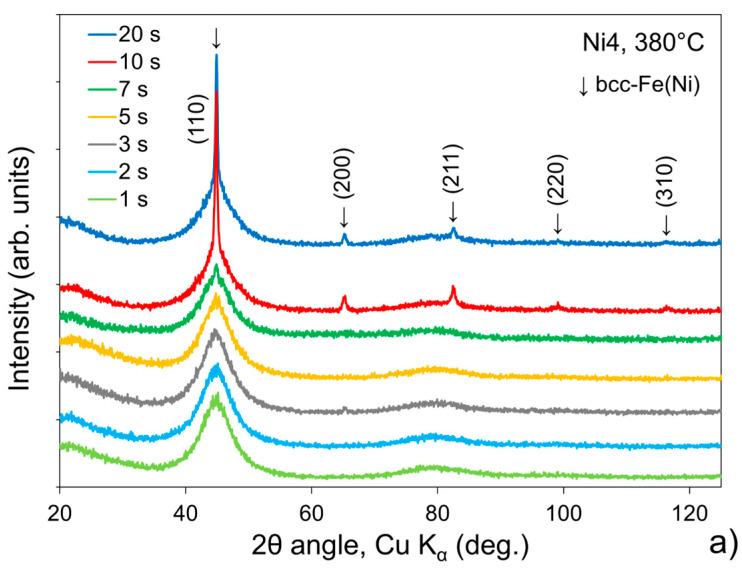

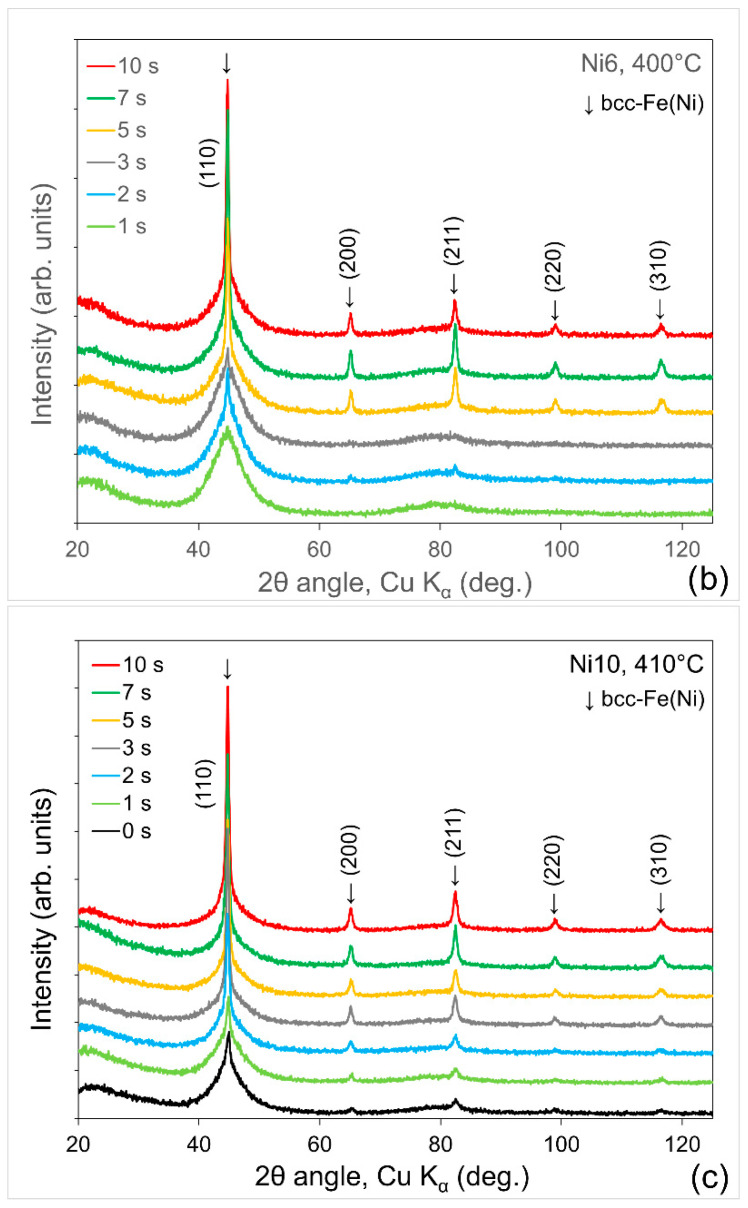
X-ray diffraction patterns of (**a**) Fe_82_Ni_4_B_14_ alloy annealed at 380 °C, (**b**) Fe_80_Ni_6_B_14_ alloy annealed at 400 °C and (**c**) Fe_76_Ni_10_B_14_ alloy annealed at 410 °C.

The structural investigation by X-ray diffraction (XRD) revealed that at lower temperatures the alloys did not crystallise immediately after reaching the isothermal stage. Amorphous structure was maintained for even up to 7 s (Ni4, 380 °C) before the first crystals were detected by XRD. This behaviour is consistent with the results obtained from the in situ measurements of resistance. Figure 4 presents the dependence of the crystalline phase volume fraction, *V*_cr_, on the temperature and duration of the isothermal stage. The *V*_cr_ = f(*T*_iso_, *t*_iso_) plots show that once crystallisation starts, it is relatively fast, and its rate decays with time. Higher temperature enhances crystallisation, because it directly controls both stages: nucleation and growth of grains. The continuous decrease in crystallisation rate over time suggests that the process is controlled mainly by diffusion, which is the principal feature of primary crystallisation. Slow diffusion is the main mechanism of preventing excessive grain growth. In the case of the investigated alloys, the diffusion-controlling component is boron, insoluble in iron. The diffusion of minority Ni atoms in the Fe matrix is slower than the self-diffusion of the majority Fe atoms, so a larger concentration of nickel is likely to slow down the grain growth.

#### 3.2.2. Transmission Electron Microscopy

**Figure 5:**
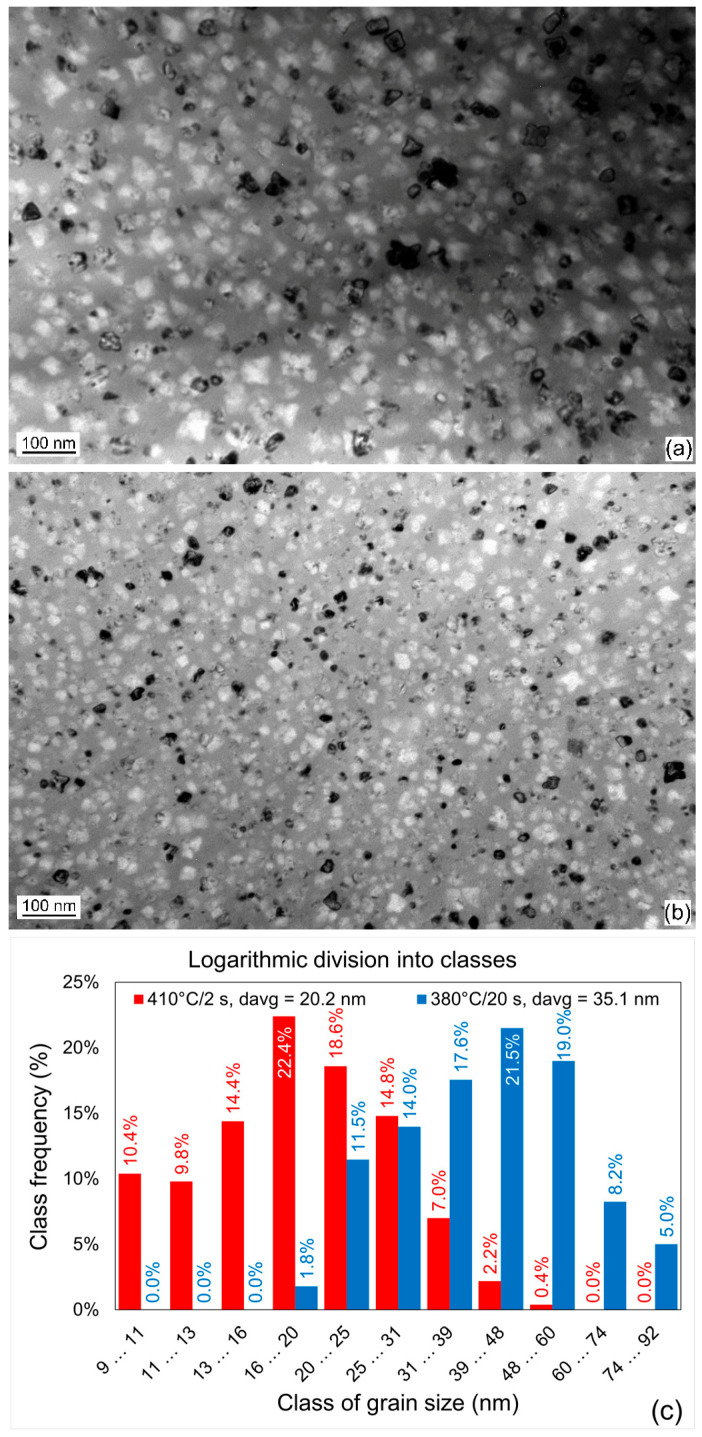
Bright-field TEM images of the Fe_76_Ni_10_B_14_ alloy annealed at (**a**) 380 °C for 20 s, (**b**) 410 °C for 2 s, and (**c**) histograms of grain size of samples (**a**,**b**).

For the analysis of the impact of heat treatment conditions on the microstructure of nanocrystalline alloys, the Ni10 alloy was taken, because it was possible to obtain nanocrystalline structure in the widest temperature range of 30 °C. Other alloys, as depicted in Figure 3, were overheated at higher temperatures, so they were less suitable for comparison. For the microstructure analysis of the Ni10 alloy, two extreme states were taken: after isothermal annealing at 380 °C for 20 s and after 410 °C for 2 s. In both samples, the bcc-Fe(Ni) phase occupies about 33% of volume, and their chemical composition is identical; therefore, the grain morphology depends only on the kinetics of nucleation and growth of the crystalline phase. Figure 5 shows the bright-field TEM images of the Ni10 alloy and the grain size distribution calculated from the image analysis. In the case of the sample annealed at 410 °C for a short time, the average grain size, *d*_avg_, was 20.2 nm, with a significant fraction of small grains. For the sample annealed at a lower temperature, the average grain size was 35.1 nm, and grains smaller than 15.8 nm were not found. The grains observed in Figure 5 are larger compared to those reported in [7], although they are still in the nanometric range and comparable with numerous examples found in the literature. The latter observation suggests that in the sample annealed at 380 °C for 20 s, nucleation was discontinued at some point, and no new grains were formed. After that point, the increase in the crystalline phase volume was due to the growth of the existing grains. This suggests that all the potential nucleation sites were exploited, and supercritical nuclei were not further created. Pre-existing nuclei or nuclei formed during incubation time were the seeds of the grains observed by TEM. The hindering of the nucleation process could also be caused by the change in boron content in the remaining amorphous matrix from the nominal 14% in the as-quenched state to about 21% after 1/3 of volume was transformed into boron-free crystals. The increase in boron content increases the crystallisation temperature [18]; therefore, the formation of new grains was suppressed. In the case of annealing at 410 °C, this temperature is likely to be sufficient to support crystallisation from the amorphous matrix.

Another reason for the smaller size of grains in the sample annealed at higher temperature is the number of grains per unit of volume. Each grain of bcc-Fe(Ni) expels boron, because B solubility in Fe is negligible. Before the front of crystallisation, atoms of B form a “cushion”, where crystallisation requires higher temperature, but boron atoms can migrate to the space around. If there are just a few grains in a certain volume, the distance between neighbours is large, boron atoms can diffuse away, and the grains can grow easily. Inversely, if the grains are numerous and close to each other, their “cushions” overlap, boron cannot be expelled away, and the grains impede each other’s growth. This is called a “soft pinning” mechanism. In the case of both investigated states, the number density of grains per unit volume was calculated from the crystalline phase’s volume fraction and the distribution of grain size, taking into account the volume-weighed correction. The number of grains in 1 cubic micrometre was equal to about 87,000 and 432,000 for the samples annealed at 380 °C for 20 s and at 410 °C for 2 s, respectively. These numbers prove that the efficiency of supercritical nuclei creation at 410 °C is approximately five times higher than that at 380 °C. Thanks to the much greater number density of grains, the soft pinning mechanism is a very important factor limiting the growth of nanocrystals.

### 3.3. Magnetic Properties

**Figure 6:**
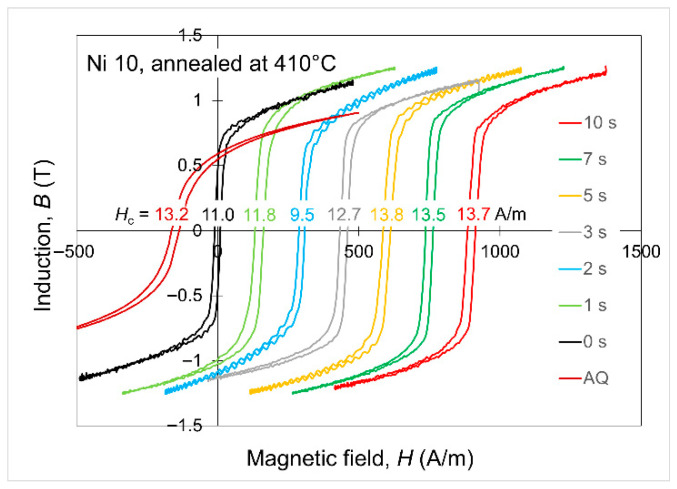
Magnetic hysteresis loops of the Fe_76_Ni_10_B_14_ alloy annealed at 410 °C. For better visibility, the loops were shifted along the *H* axis.

The magnetic properties of all the alloys after annealing were studied by the measurement of the hysteresis loop. As an example of the set of recorded hysteresis loops, Figure 6 presents the loops of the Ni10 alloy annealed at 410 °C, with isotherm duration from 0 s to 10 s. The loop for the as-quenched (AQ) state was added for comparison.

**Figure 7:**
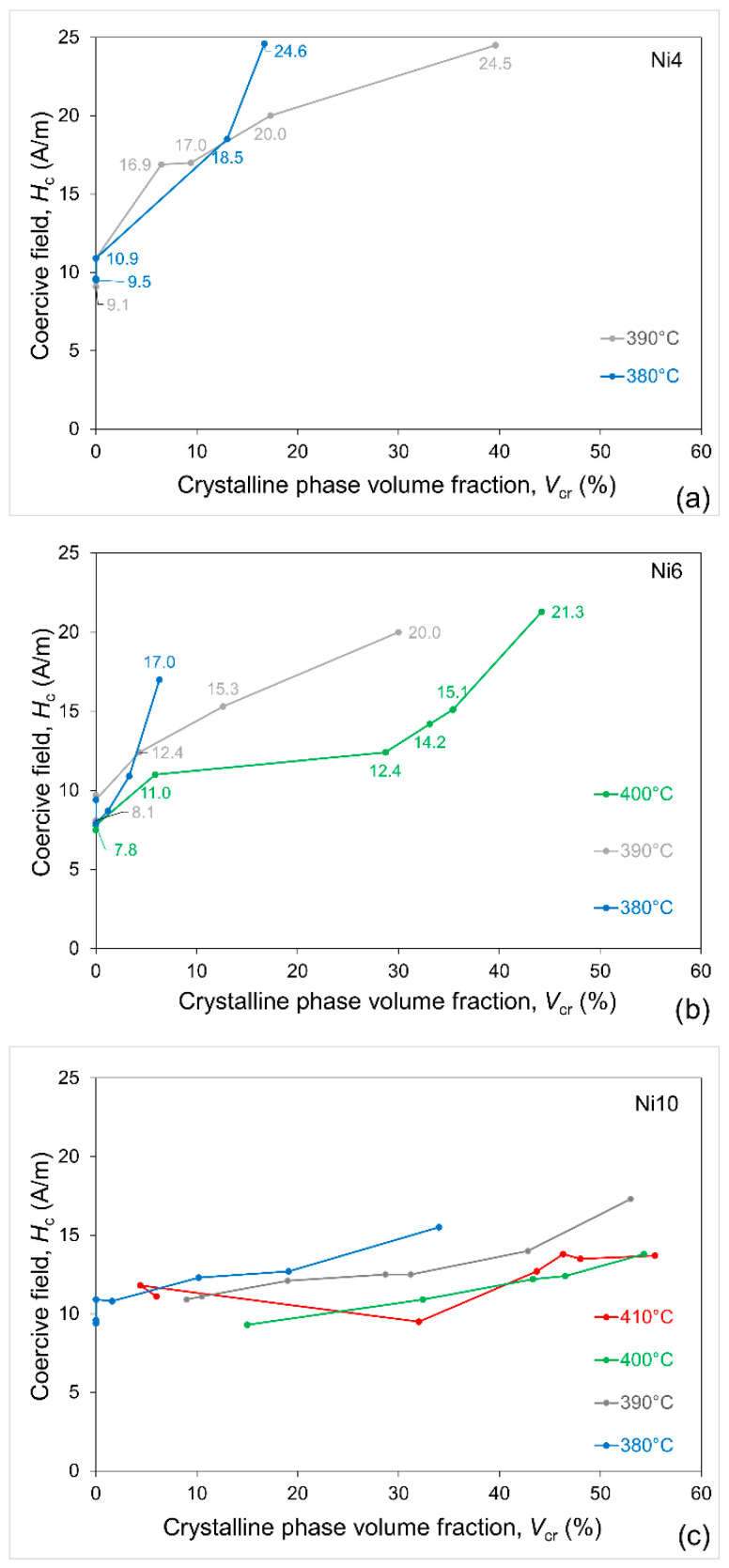
Dependence of coercive field on crystalline volume fraction for (**a**) Fe_82_Ni_4_B_14_, (**b**) Fe_80_Ni_6_B_14_ and (**c**) Fe_76_Ni_10_B_14_ alloys annealed at various temperatures.

From the hysteresis loops, the coercive field was read as the measure of magnetic softness. The dependence of the coercive field on the crystalline phase volume fraction for all the alloys is depicted in Figure 7. In all the cases, consistently, *H*_c_ was lower if the alloys were annealed at higher temperature. Since smaller grains are the reason for magnetic softness [6], the results of magnetic measurements are in accordance with the conclusions from the TEM image analysis and from the XRD line broadening, which showed that higher annealing temperature yields finer grains. Apart from the microstructural aspect, the observed better magnetic softness of the alloys with Ni addition is the effect of the lower magnetocrystalline anisotropy, *K*_1_, of the Fe-Ni alloys due to the presence of nickel [5].

### 3.4. Brittleness Assessment

**Figure 8:**
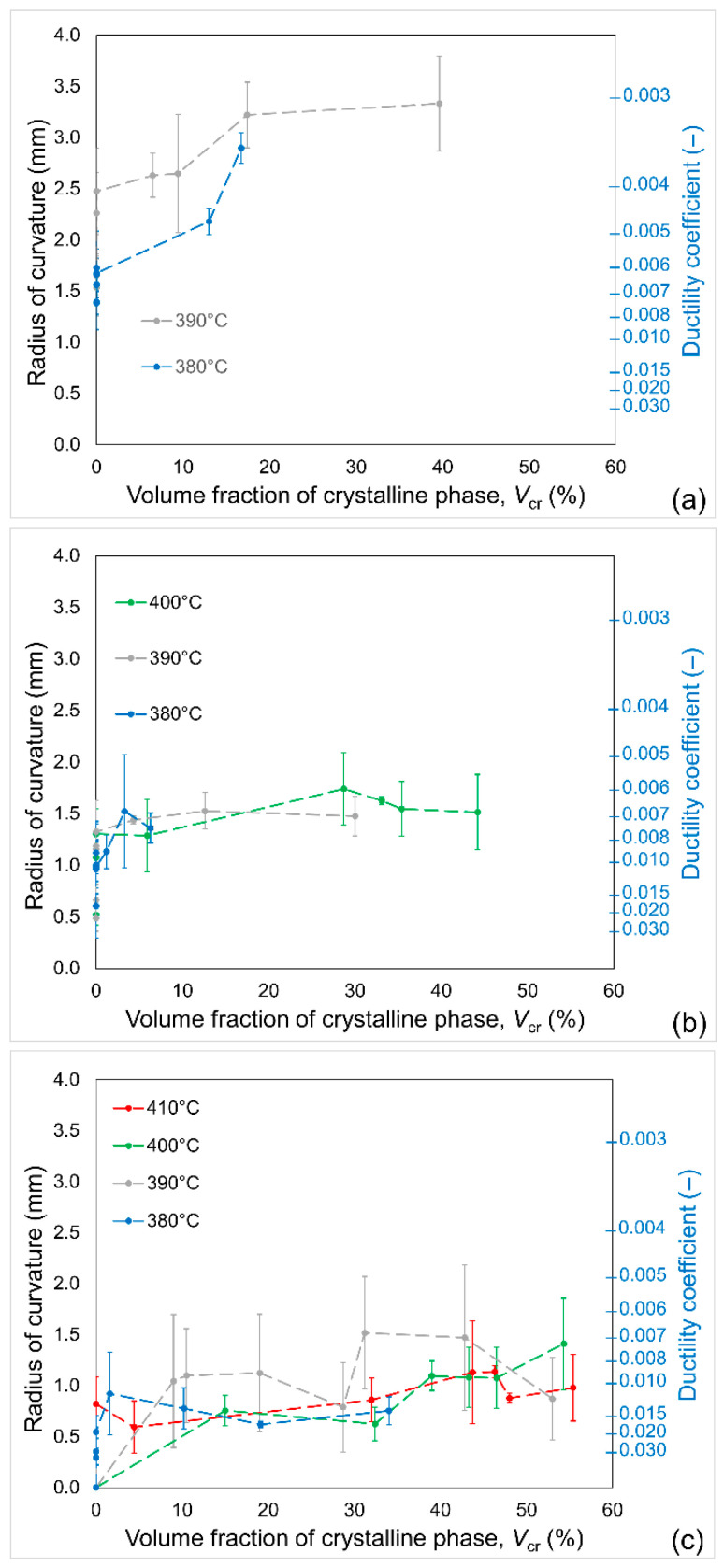
Dependence of radius of curvature of (**a**) Fe_82_Ni_4_B_14_, (**b**) Fe_80_Ni_6_B_14_ and (**c**) Fe_76_Ni_10_B_14_ alloys annealed at various temperatures. Error bars show the standard deviation.

The ribbons after annealing were tested for brittleness by 180° bending. Each sample was broken several times in different places along its length to improve the statistics, because the samples had a tendency to break prematurely. The individual results, if outstanding from the other results for a sample, were rejected, and the average of non-scattered values was presented. The dependence of brittleness on the crystalline phase volume fraction is presented in Figure 8. Since the ductility coefficient, *λ*, is impractical, brittleness is expressed as the radius of curvature at fracture. The main observation from the plots of brittleness is that the addition of nickel significantly improves the ductility of the investigated Fe-Ni-B alloys. In addition, after the bcc-Fe(Ni) phase occupies about 15–20% of volume, the brittleness reaches a stable level and the alloys do not lose more ductility. For substantially crystallised alloys, one can observe that their brittleness does not depend on the annealing temperature, which is important for the selection of rapid annealing parameters that allow optimisation of the magnetic and mechanical properties of nanocrystalline Fe-Ni-B alloys. This observation means that for iron-based nanocrystalline–amorphous alloys obtained by ultra-rapid annealing, the temperature of annealing is of less importance, if brittleness is of concern, so adjustment of annealing parameters should take into account the magnetic properties.

## 4. Conclusions

- Ultra-rapid annealing of Fe-Ni-B alloys leads to a significant increase in onset crystallisation temperature, which exceeds the glass transition temperature; therefore, crystallisation occurs in the supercooled liquid region.
- Analysis of changes in the resistance of the isothermally annealed samples revealed that, prior to crystallisation, there is an incubation period. The addition of nickel shortens the incubation period.
- Increasing the temperature and shortening time of isothermal annealing yields better magnetic softness of nanocrystalline–amorphous alloys due to the more intense nucleation of the crystalline phase, the shorter time of grain growth and, eventually, a finer microstructure.
- The presence of nickel reduces the size of nanocrystals and yields a lower coercive field.
- The applied ultra-rapid Joule heating technology makes it possible to obtain a nanocrystalline structure. It is possible to control the microstructure and magnetic properties of Fe-Ni-B alloys by adjusting the heat treatment parameters.
- The addition of nickel significantly improved the ductility of Fe-Ni-B ribbons after nanocrystallisation. After reaching ~15–20 vol.% of the crystalline phase, brittleness stabilised and became independent of annealing temperature.

## Figures and Tables

**Table 1:** Incubation time, *τ*_0_, in seconds for the Fe_86-x_Ni_x_B_14_ alloys subjected to ultra-rapid annealing with heating rate of 100 °C.

Annealing Temperature (°C)	Fe_82_Ni_4_B_14_	Fe_80_Ni_6_B14	Fe_76_Ni_10_B_14_
380 °C	2.68	5.65	2.73
390 °C	1.54	1.96	1.05
400 °C	1.05	1.66	0.39
410 °C	overheated	0.79	0.22

## Data Availability

Data shall be made available on request.

## Abbreviations

The following abbreviations are used in this manuscript:

URA	Ultra-rapid annealing
XRD	X-ray diffraction
TEM	Transmission electron microscopy
